# The quality of mental health care delivered to patients with schizophrenia and related disorders in the Italian mental health system. The QUADIM project: a multi-regional Italian investigation based on healthcare utilisation databases

**DOI:** 10.1017/S2045796022000014

**Published:** 2022-02-14

**Authors:** Antonio Lora, Matteo Monzio Compagnoni, Liliana Allevi, Angelo Barbato, Flavia Carle, Barbara D'avanzo, Teresa Di Fiandra, Lucia Ferrara, Andrea Gaddini, Melania Leogrande, Alessio Saponaro, Salvatore Scondotto, Valeria D. Tozzi, Simona Carbone, Giovanni Corrao

**Affiliations:** 1 Department of Mental Health and Addiction Services, ASST Lecco, Lecco, Italy; 2 National Centre for Healthcare Research and Pharmacoepidemiology, University of Milano-Bicocca, Milan, Italy; 3 Unit of Biostatistics, Epidemiology and Public Health, Department of Statistics and Quantitative Methods, University of Milano-Bicocca, Milan, Italy; 4 Unit for Quality of Care and Rights Promotion in Mental Health, Istituto di Ricerche Farmacologiche Mario Negri IRCCS, Milano, Italy; 5 Center of Epidemiology and Biostatistics, Polytechnic University of Marche, Ancona, Italy; 6 Ministry of Health, Rome, Italy; 7 Centre of Research on Health and Social Care Management, SDA Bocconi School of Management (Bocconi University) Milan, Milan, Italy; 8 Agency for Public Health, Lazio Region, Rome, Italy; 9 Health and Social Policies, Emilia-Romagna Region, Bologna, Italy; 10 Department of Health Services and Epidemiological Observatory, Regional Health Authority, Sicily Region, Palermo, Italy; 11 Department of Health Planning, Italian Health Ministry, Rome, Italy

**Keywords:** Clinical indicators, healthcare utilisation database, quality of mental healthcare, schizophrenia and related disorders

## Abstract

**Aims:**

To evaluate the quality of mental health care delivered to patients with schizophrenia and related disorders taken-in-care by mental health services in four Italian regions (Lombardy, Emilia-Romagna, Lazio, Sicily).

**Methods:**

Thirty-one clinical indicators concerning accessibility, appropriateness, continuity and safety were defined and estimated using healthcare utilisation (HCU) databases, containing data on mental health treatments, hospital admissions, outpatient interventions, lab tests and drug prescriptions.

**Results:**

A total of 70 586 prevalent patients with schizophrenia and related disorders treated in 2015 were identified, of whom 1752 were newly taken-in-care by the facilities of regional mental health services. For most patients community care was accessible and moderately intensive. However, care pathways were not implemented based on a structured assessment and only half of the patients received psychosocial treatments. One patient out of ten had access to psychological interventions and psychoeducation. Activities specifically addressed to families involved a third of prevalent patients and less than half of new patients. One patient out of six was admitted to a community residential facility, and one out of ten to a General Hospital Psychiatric Ward (GHPW); higher values were identified in new cases. In general hospitals, few patients had a length of stay (LoS) of more than 30 days, while one-fifth of the admissions were followed by readmission within 30 days of discharge. For two-thirds of patients, continuity of community care was met, and six times out of ten a discharge from a GHPW was followed by an outpatient contact within 2 weeks. For cases newly taken-in-care, the continuity of community care was uncommon, while the readiness of outpatient contacts after discharge was slightly more frequent. Most of the patients received antipsychotic medication, but their adherence to long-term treatment was low. Antipsychotic polytherapy was frequent and the control of metabolic side effects was poor. The variability between regions was high and consistent in all the quality domains.

**Conclusions:**

The Italian mental health system could be improved by increasing the accessibility to psychosocial interventions, improving the quality of care for newly taken-in-care patients, focusing on somatic health and mortality, and reducing regional variability. Clinical indicators demonstrate the strengths and weaknesses of the mental health system in these regions, and, as HCU databases, they could be useful tools in the routine assessment of mental healthcare quality at regional and national levels.

## Introduction

The 1978 Italian reform of psychiatric services initiated the closure of psychiatric hospitals encouraging the development of a widespread and complex network of community mental health facilities (Lora, 2009; Lora *et al*., 2012). Although, in recent years, many countries significantly invested in community-based mental health care, the Italian community model was implemented earlier than in the rest of the world, and relevant efforts were made to move away from the institutional model. Therefore, Italy can be viewed as a laboratory to assess the quality of mental health care delivered in a community-oriented system, especially for severe mental disorders (Barbui *et al*., 2018; Kilbourne *et al*., 2018). Two Italian National Health Plans made the same recommendations for implementing and managing mental health facilities all over the country. However, as the regions were responsible for managing the transition, there is wide variability across regions in the amount of resources devoted to community-based psychiatric care, and the range of services provided still is a cause of concern (Ferrannini *et al*., 2014).

Quality of care has been defined as the *degree to which health services for individuals and population are effective, safe and people-centred* (WHO, 2018). The core dimensions of health quality may be articulated in sub-dimensions (i.e. access, appropriateness, continuity, timeliness, efficiency and equity) that could be used to evaluate the quality of care (Spaeth-Rublee *et al*., 2010; OECD and European Observatory on Health Systems and Policies, 2019). Therefore, and in line with available evidence and best practice, clinical indicators for (i) measuring quality of care sub-dimensions, (ii) allowing benchmarking, (iii) establishing priorities for quality improvement and (iv) supporting accountability in mental health care (Lora *et al*., 2017) have been developed (Mainz, 2003; Lauriks *et al*., 2012; Samartzis and Talias, 2019).

Although initiatives based on clinical indicators for assessing the quality of mental health care have been developed by transnational-organisations (e.g. promoted by the OECD (OECD, 2021), the WHO (Rotar *et al*., 2016; WHO, 2020)), the single countries of northern Europe (Baandrup *et al*., 2016; NICE, 2021), America (e.g. the Canadian Institute for Health Information (Smith *et al*., 2018; CIHI, 2021), the National Quality Forum (NQF, 2021; SAMHSA, 2021)) and Oceania (Australian Institute of Health and Welfare, 2021; Health Quality& Safety Commission New Zealand, 2021), there is still no widespread practice of measuring the quality of mental care. Not surprisingly, the OECD stated that the quality of care for those with mental health disorders *will continue to trail behind that for other diseases until appropriate indicators are used to measure quality, and appropriate data are collected* (OECD, 2014).

Against this background, the QUADIM project (an Italian multi-regional project called ‘Clinical pathways in patients with severe mental disorders in Italy’) was funded by the Italian Ministry of Health (MoH, Health Prevention Department) to evaluate the quality of healthcare pathways for patients with severe mental disorders. Specifically, this paper aims to assess the quality of care routinely delivered to prevalent and newly taken-in-care patients with schizophrenia and related disorders using clinical indicators to identify the strengths and weaknesses of the mental health systems of three Italian regions (Lombardy, Emilia-Romagna, Lazio) and one province (Palermo, Sicily).

## Methods

### Setting and data sources

In Italy, mental healthcare is provided by public Departments of Mental Health (DMHs), organised into a network of community services including Community Mental Health Centres (CMHCs), General Hospital Psychiatric Wards (GHPWs), Day-Care Centres (DCs) and Community Residential Facilities (CRFs). Private healthcare providers deliver day-care and residential care in conjunction with public DMHs.

The data for the QUADIM project were retrieved retrospectively from the healthcare utilisation (HCU) databases of three Italian regions, located in the northwest (Lombardy), northeast (Emilia-Romagna), centre (Lazio), and in one province, located in the south (Palermo) (Corrao *et al*., 2021). All Italian citizens have equal access to health care as part of the National Health Service (NHS), and an automated system of HCU databases allows each region to manage healthcare locally. HCU data include a range of information on residents who receive NHS care: discharges from public or private hospitals, outpatient drug prescriptions, specialist visits and diagnostic exams, all reimbursable by the NHS. In addition, a national specific information system concerning psychiatric care is implemented by the regional DMHs and private facilities accredited by the NHS (the ‘Italian Mental Health Information System’). This system collects sociodemographic information, ICD-10 or ICD-9-CM diagnoses, and records all treatments provided (outpatient and home visits, day-care attendances, admissions to hospital and CRFs) for all patients receiving mental health care. The entire list of interventions provided by mental health services is reported in online Supplementary Table S1. As a unique identification code is used for all databases within each region, it was possible to link HCU databases through a record-linkage procedure, enabling the study of the complete care pathway of NHS beneficiaries. For privacy issues, identification codes are automatically anonymised. Details of HCU databases use in the field of mental health have been reported in more details elsewhere (Corrao *et al*., 2015, 2021; Lora *et al*., 2016).

### Harmonisation and data processing

Although HCU databases do not substantially differ across all regions, a between-regions data harmonisation was performed, allowing consistent data extraction processes (e.g. information was uniformly encoded by using the same names, values and formats). Anonymised data were extracted and processed locally using a common Statistical Analysis System (SAS) program developed by one of the authors (MMC), according to the protocol previously shared and approved by the project working groups. Diagnostic and therapeutic codes used for drawing records and fields from databases are reported in online Supplementary Table S2.

### Cohort selection

The target population consisted of all NHS beneficiaries residing in Lombardy, Emilia-Romagna, Lazio and Palermo aged 18–65 (Corrao *et al*., 2021). Those with a diagnosis of schizophrenia and related disorders who, from January 2015 to December 2015, had at least one contact with a DMH were identified. These patients were labelled as prevalent cases, and the date of their first contact with a DMH during the recruitment was recorded as the index date. Then, to include the cohort of newly taken-in-care patients (e.g. those with first-lifetime diagnosis of schizophrenia and related disorders known to the NHS), prevalent cases were excluded if they (i) received a diagnosis of schizophrenia and related disorders at any time before the index date, (ii) experienced any hospital admission to a psychiatric ward, and/or (iii) received at least two consecutive prescriptions for antipsychotic drugs within the two years before the index date. Because there is some residual uncertainty regarding the ability of this algorithm to identify new diagnoses, the latter study cohort was restricted to patients aged 18–40 years (Corrao *et al*., 2021).

Members of both cohorts accumulated person-years of follow-up starting from the index date until one year after the index date (endpoint of follow-up). Those who did not reach at least one year of follow-up were excluded.

### Clinical indicators

Thirty-one quality indicators were jointly designed by two multidisciplinary expert groups, both funded by the Italian MoH (Corrao *et al*., 2019, 2021). The process of building indicators suitable for the quality assessment of care in schizophrenic disorders has been described elsewhere (Lora *et al*., 2016). Those indicators were designed starting from evidence-based recommendations tailored to community care goals produced with the agreement of the Italian MoH and regional governments (Conferenza Unificata Stato-Regioni, 2014), and considering the guidelines developed by the American Psychiatric Association (Keepers *et al*., 2020) and the National Institute for Clinical Excellence (National Collaborating Centre for Mental Health (UK), 2014) as a milestone for the treatment of schizophrenia spectrum disorders. Recommendations, and the derived indicators, identified the interventions needed by essential clinical pathways for the treatment of severe mental illness and for their monitoring. Finally, the 31 clinical indicators identified were classified into three groups, each one related to one of the above-defined core dimensions of health quality: accessibility and appropriateness (*n* = 22), continuity (*n* = 5) and safety (*n* = 4) of the mental health care.

The need to retrieve the indicators from regional HCU databases led to the identification of process indicators, except for mortality and admission to GHPWs, as outcome indicators.

### Statistical analysis

Prevalence and incidence rates, and mean values of the indicators were computed for each region/province and for the whole aggregated sample. As calculations were separately performed within each considered region, summarised estimates were obtained by pooling aggregated regional data.

The hypothesis of homogeneity among regional estimates was tested using (i) the *χ*^2^ test for clinical indicators expressed as proportions or (ii) the one-way analysis of variance (ANOVA) procedure for indicators expressed as the mean number of interventions per person-years of follow-up. Furthermore, the level of heterogeneity of estimates between regions was measured with the *I*^2^ statistics (the proportion of between-region variability due to heterogeneity) (Higgins *et al*., 2003).

The prescriptions of antipsychotics dispensed to patients during the follow-up were identified and used to evaluate persistence with the recommended pharmacotherapy. The duration of each prescription was calculated by dividing the total amount of prescribed drugs by the defined daily dose. Prescriptions were considered ‘consecutive’ if the interval between the end of one prescription and the start of the following one was less than 90 days, and ‘interrupted’ otherwise; interrupted prescriptions were considered to lead to discontinuation of treatment. All outpatient contacts provided by CMHCs or DCs were identified to evaluate the persistence with community care, and a patient was considered persistent if they experienced at least one community contact every 90 days in the 365 days after the first contact. The time spent in hospital and residential wards was considered continuity of care.

The expected number of deaths was calculated by grouping male and female patients by age (in 5-year age-interval groups) and multiplying the number of patients in each group by the corresponding age- and gender-specific mortality rates among the general population of each region in the year 2015 (source: Italian Institute of Statistics). The standardised mortality ratio (SMR), which gives the ratio between observed and expected deaths, was calculated. The corresponding 95% CI were calculated by assuming that the observed number of deaths followed a Poisson distribution.

All the analyses were separately performed for each of the two considered cohorts and for each region, using the SAS Software (version 9.4; SAS Institute, Cary, NC, USA) and Microsoft Excel (Version 2019 16.0.6742.2048). For all hypotheses tested, two-tailed *p*-values <0.05 were considered significant.

## Results

**Fig. 1.**
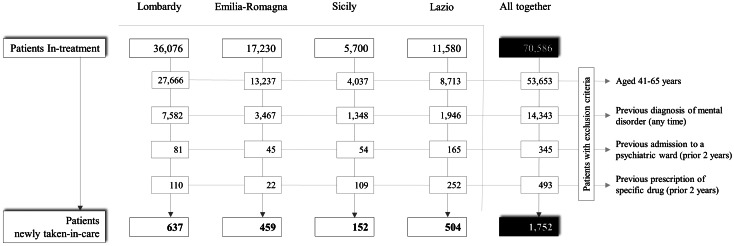
Flow-chart of inclusion and exclusion criteria for the eligibility of patients newly taken-in-care with schizophrenia and related disorders in three regions (Lombardy, Emilia-Romagna, Lazio) and one province (Palermo), and in the whole Italian sample. Italy, QUADIM-MAP projects, Italy, 2015–2016.

The cohort selection process is shown in Fig. 1. A total of 70 586 prevalent patients with a diagnosis of schizophrenia and related disorders were identified, while the cases newly taken-in-care amounted to 1752. The sociodemographic and diagnostic characteristics of the two study cohorts are shown in online Supplementary Tables S3 and S4.

Age-standardised rates (per 10 000 inhabitants aged 18–65) of prevalence for schizophrenia and related disorders ranged from 23.5 (Lazio) to 54.6 (Palermo), while the overall prevalence rate was 39.2. Age-standardised rates for incidence (per 10 000 inhabitants aged 18–40) ranged from 2.5 (Lombardy) to 4.2 (Emilia-Romagna), while the overall rate was 3.1. The estimated values for the clinical indicators are shown in Table 1 for prevalent patients and in Table 2 for those newly taken-in-care.

**Table 1.** Estimated values for the clinical indicators for prevalent patients with schizophrenia and related disorders treated by DMHs of four Italian areas (Lombardy, Emilia Romagna and Lazio Regions and Province of Palermo) and in the whole sample

		Lombardy (*n* = 36 076)	Emilia-Romagna (*n* = 17 230)	Palermo (*n* = 5700)	Lazio (*n* = 11 580)	Whole sample (*n* = 70 586)	*I*^2^ ^a^
	Age-, gender-standardised treated prevalence rate (×10 000)	43.4	46.1	54.6	23.5	39.2	–
Accessibility and appropriateness of mental health care							
1	Patients with at least one contact in CMHCs or DCs	92.7%	97.6%	81.1%	91.6%	92.8%*	99
2	Mean number of patients' outpatient contacts at CMHCs *(per year)*	24.4	44.1	10.6	22.0	27.7*	99
3	Mean number of patients' outpatient psychiatric visits *(per year)*	4.8	4.4	4.4	6.1	4.9*	99
4	Patients with standardised assessments using tests	3.0%	2.2%	0.7%	1.9%	2.4%*	98
5	Patients delivered home care *(at least one home visit)*^b^	19.0%	NA	10.7%	20.0%	18.4%*	99
6	Patients treated with psychosocial interventions	49.8%	58.5%	60.5%	54.3%	53.5%*	99
7	Mean number of psychosocial interventions at CMHCs *(per year, in patients with at least one intervention)*	13.8	22.9	5.3	9.4	14.7*	99
8	Patients treated with psychoeducation *(at least one session)*^c^	3.1%	6.1%	5.9%	NA	4.2%*	99
9	Mean number of psychoeducation sessions *(per year, in patients with at least one session)*^c^	4.5	9.2	2.0	NA	4.9*	99
10	Patients treated with psychotherapy *(at least one session)*	7.9%	4.4%	9.3%	13.6%	8.1%*	99
11	Mean number of psychotherapy sessions *(per year, in patients with at least one session)*	8.2	8.2	6.3	9.7	8.4*	95
12	Patients with at least one outpatient carers' contact	34.5%	39.5%	57.5%	26.3%	36.2%*	99
13	Mean number of interventions specifically addressed to patients' family members *(per year)*	1.4	2.0	2.5	0.8	1.5*	99
14	Patients treated with antipsychotic agents	79.0%	90.0%	70.3%	77.9%	80.8%*	99
15	Patients treated with antipsychotic polytherapy for more than 30 days *(in patients treated with antipsychotics)*	31.5%	65.7%	14.3%	38.2%	40.7%*	99
16	Patients with at least one admission in residential facilities	16.6%	19.0%	2.5%	10.3%	15.0%*	99
17	Mean number of days spent in residential facilities *(in patients with at least one admission)*	156.2	191.9	223.2	172.5	170.8*	98
18	Patients with at least one admission in GHPW	10.7%	10.1%	13.1%	8.0%	10.3%*	97
19	Mean number of days spent in GHPW *(in patients with at least one admission)*	28.3	26.9	23.9	49.6	31.5*	99
20	Admissions with a length of stay in GHPW higher than 30 days	9.7%	6.9%	5.2%	7.6%	8.4%*	92
21	Unplanned re-admissions in GHPW within 7 days^d^	9.3%	10.4%	5.9%	13.6%	9.7%	97
22	Unplanned re-admissions in GHPW within 30 days^d^	20.0%	21.2%	16.6%	23.3%	20.2%	84
Continuity of mental health care							
23	Patients with continuous community care	57.2%	74.3%	45.1%	66.7%	62.3%*	99
24	GHPW discharges followed by any mental health outpatient contact within 14 days^d^	58.3%	73.7%	47.3%	66.1%	61.2%*	98
25	GHPW discharges followed by an outpatient psychiatric visit within 14 days^d^	36.5%	34.2%	35.6%	46.1%	37.1%*	94
26	GHPW discharges followed by home care within 14 days^b^^,^^d^	8.3%	NA	2.1%	6.9%	5.7%*	97
27	Patients persistent with antipsychotic therapy	44.2%	52.8%	68.2%	52.0%	49.8%*	99
Safety of mental health care							
28	Patients monitored for hyperglycaemia and hyperlipidaemia *(in patients treated with antipsychotics)*	28.6%	30.8%	29.0%	18.8%	27.7%*	99
29	Patients monitored for hyperlipidaemia *(in patients treated with antipsychotics)*	30.0%	32.2%	31.0%	19.6%	29.0%*	99
30	Patients monitored for hyperglycaemia *(in patients treated with antipsychotics)*	45.4%	44.4%	45.1%	41.4%	44.5%*	93
31	Mortality (SMR), and relative 95% CI	1.6 (1.5-1.7)	1.6 (1.5-1.7)	2.6 (2.2-2.9)	1.9 (1.7-2.1)	1.7 (1.6-1.8)*	–

DMH, Department of Mental Health; CMHC, Community Mental Health Centres; DC, Day-Care Centres; GHPW, General Hospital Psychiatric Wards; SMR, standardised mortality ratio.

Italy, QUADIM-MAP projects, Italy, 2015–2016.

a Values of *I*^2^ are percentages.

b Information for Emilia-Romagna Region was not available for this clinical indicator, which was calculated on the 53 356 remaining patients.

c Information for Lazio Region was not available for this clinical indicator, which was calculated on the 59 006 remaining patients.

d After a previous hospital admission in GHPW (statistical unit).

*p-value < 0.05 for test of homogeneity among indicators' regional estimates.

**Table 2.** Estimated values for the clinical indicators for patients with schizophrenia and related disorders newly taken-in-care by DMHs of four Italian areas (Lombardy, Emilia Romagna and Lazio Regions and Province of Palermo) and in the whole sample

		Lombardy (*n* = 637)	Emilia-Romagna (*n* = 459)	Palermo (*n* = 152)	Lazio (*n* = 504)	Whole sample (*n* = 1752)	*I*^2^^a^
	Age-, gender-standardised incidence rate (×10 000)	2.5	4.2	4.1	3.2	3.1	–
Accessibility and appropriateness of mental health care							
1	Patients with at least one contact in CMHCs or DCs	87.1%	92.2%	82.2%	86.3%	87.8%*	96
2	Mean number of patients' outpatient contacts at CMHCs *(per year)*	20.7	31.7	14.4	16.8	22.0*	95
3	Mean number of patients' outpatient psychiatric visits *(per year)*	6.4	6.2	5.2	6.6	6.3*	59
4	Patients with standardised assessments using tests	10.7%	13.7%	1.3%	7.7%	9.8%*	95
5	Patients delivered home care *(at least one home visit)*^b^	10.4%	NA	7.9%	9.5%	9.7%	2
6	Patients treated with psychosocial interventions	57.0%	63.2%	67.8%	54.8%	58.9%*	77
7	Mean number of psychosocial interventions at CMHCs *(per year, in patients with at least one intervention)*	13.3	13.9	8.6	8.1	11.6*	89
8	Patients treated with psychoeducation *(at least one session)*^c^	7.8%	13.7%	8.6%	NA	10.1%*	78
9	Mean number of psychoeducation sessions *(per year, in patients with at least one session)*^c^	4.9	5.3	3.0	NA	4.9	96
10	Patients treated with psychotherapy *(at least one session)*	27.4%	16.8%	27.6%	21.4%	21.5%*	73
11	Mean number of psychotherapy sessions *(per year, in patients with at least one session)*	9.6	8.2	6.8	8.3	8.6	82
12	Patients with at least one outpatient carers' contact	47.9%	50.8%	63.8%	28.2%	44.3%*	97
13	Mean number of interventions specifically addressed to patients' family *(per year)*	2.6	3.0	3.9	1.0	2.4*	97
14	Patients who started antipsychotic treatment	72.2%	81.3%	57.2%	59.1%	69.5%*	95
15	Patients treated with antipsychotic polytherapy for more than 30 days *(in patients treated with antipsychotics)*	32.2%	57.4%	13.8%	30.2%	38.1%*	97
16	Patients with at least one admission in residential facilities	26.1%	27.9%	2.6%	12.7%	20.8%*	98
17	Mean number of days spent in residential facilities *(in patients with at least one admission)*	64.3	60.2	118.6	107.8	71.8*	96
18	Patients with at least one admission in GHPW	20.6%	23.7%	20.4%	13.3%	19.3%*	85
19	Mean number of days spent in GHPW *(in patients with at least one admission)*	33.9	31.1	14.5	38.6	32.3*	98
20	Admissions with a length of stay in GHPW higher than 30 days	9.5%	6.2%	0.0%	1.4%	6.4%*	72
21	Unplanned re-admissions in GHPW within 7 days^d^	12.6%	13.1%	12.5%	7.2%	11.9%*	10
22	Unplanned re-admissions in GHPW within 30 days^d^	20.1%	22.8%	17.5%	8.7%	19.0%	69
Continuity of mental health care							
23	Patients with continuous community care	49.0%	38.4%	44.4%	52.6%	47.9%*	99
24	GHPW discharges followed by any mental health outpatient contact within 14 days^d^	58.8%	71.0%	47.5%	75.4%	64.2%*	79
25	GHPW discharges followed by an outpatient psychiatric visit within 14 days^d^	45.2%	39.3%	45.0%	62.3%	45.9%*	71
26	GHPW discharges followed by home care within 14 days^b^^,^^d^	5.5%	NA	0.0%	2.9%	2.9%	8
27	Patients persistent with antipsychotic therapy	37.8%	44.0%	40.2%	41.9%	40.9%	12
Safety of mental health care							
28	Patients monitored for hyperglycaemia and hyperlipidaemia *(in patients treated with antipsychotics)*	10.2%	14.2%	4.6%	8.7%	10.7%*	74
29	Patients monitored for hyperlipidaemia *(in patients treated with antipsychotics)*	10.4%	26.5%	4.6%	9.1%	14.6%	94
30	Patients monitored for hyperglycaemia *(in patients treated with antipsychotics)*	27.4%	14.7%	14.9%	19.5%	20.7%	86

DMH, Department of Mental Health; CMHC, Community Mental Health Centres; DC, Day-Care Centres; GHPW, General Hospital Psychiatric Wards; SMR, standardised mortality ratio.

Italy, QUADIM-MAP projects, Italy, 2015–2016.

a Values of *I*^2^ are percentages.

b Information for Emilia-Romagna Region was not available for this clinical indicator, which was calculated on the 53 356 remaining patients.

c Information for Lazio Region was not available for this clinical indicator, which was calculated on the 59 006 remaining patients.

d After a previous hospital admission in GHPW (statistical unit).

* *p*-value < 0.05 for test of homogeneity among indicators' regional estimates.

As far as the *accessibility and appropriateness of community care* are concerned, about nine out of ten prevalent patients had at least one outpatient or DC intervention. On average, each patient with schizophrenia and related disorders received 27.7 outpatient contacts per year with MH professionals. The mean number of patients' outpatient psychiatric visits was 4.9 per year. Newly taken-in-care patients had a mean number of outpatient contacts and psychiatric visits of 22 and 6.3 contacts per year, respectively. Outreach programmes, like home visits, reached about two patients out of ten among prevalent cases and one out of ten among the ones newly taken-in-care. Structured assessment of clinical and psychosocial problems was infrequent. Only a tenth of the cases newly taken-in-care received this assessment, while the percentage was 2.4% in prevalent cases. About 54 and 59% of prevalent and new cases, respectively, received at least one psychosocial treatment (i.e. psychological, psychoeducational and rehabilitative interventions). The mean number of psychosocial interventions per year for prevalent cases and for the new ones was 14.7 and 11.6, respectively. Access to psychoeducation sessions was infrequent among the new cases and the prevalent ones, while the mean number of psychoeducational sessions per year was around five for both cohorts. Concerning psychological interventions, about a tenth of prevalent patients received at least one psychotherapy intervention, with a mean of 8.4 attending psychotherapy sessions per year. However, 21.5% of new cases started a psychotherapy treatment, with a mean of 8.6 sessions per year. More than one-third of the families of prevalent cases were addressed by specific interventions, whereas in families of newly taken-in-care patients, this proportion was almost 45%. Patients newly taken-in-care showed a mean of 2.4 interventions specifically addressed to patients' family members per year, being this mean equal to 1.5 in the prevalent cohort.

Regarding the recommended drug therapy, eight out of ten patients of the prevalent cohort were dispensed at least one prescription of antipsychotics. Only the 70% of patients newly taken-in-care started antipsychotic drugs treatment during the first year after the diagnosis. The proportion of patients who received antipsychotic polytherapy for more than 30 days was around 40% for both prevalent and new cases.

One prevalent patient out of six experienced at least one admission to CRF, while this proportion was one out of five among the cohort of patients newly taken-in-care. Prevalent patients admitted to CRF facilities spent a mean number of 171 days. The mean for new cases was 72. Concerning hospital care for acute events, only a tenth of prevalent patients experienced at least one hospital admission in GHPWs; among new cases this proportion was 19%. Where admissions to GHPWs are concerned, prevalent and newly taken-in-care patients showed a mean length of stay (LoS) of 31 and 32 days, respectively. The proportion of GHPW admissions with a LoS longer than 30 days was low in both cohorts. About one out of ten admissions to GHPWs was followed by an early readmission within 7 days after discharge, doubling within 30 days, in both samples.

Considering the dimension of the *continuity of care*, about two-thirds of prevalent cases received continuous community care (i.e. at least one community contact every 90 days in the 365 days after the first contact in the year), this proportion being 48% among the ones newly taken-in-care. The recommended continuity of care between GHPW and CMHCs (i.e. at least one CMHC contact within 14 days following GHPW discharge) was achieved for prevalent cases in six discharges out of ten, considering all MH professionals' contacts in CMHCs, and in four discharges out of ten, limited to psychiatric visits. These proportions for new cases were 64 and 46%, respectively. Instead, home care within 2 weeks after GHPW admission was provided in one discharge out of 20 considering prevalent patients, and, in new cases, it was even rarer. As far as the continuity of antipsychotic therapy is concerned, only a half of the prevalent cases in treatment with antipsychotic drugs were persistent. Among the newly taken-in-care patients who started antipsychotic therapy, only four out of ten did not interrupt the treatment during the follow-up.

Concerning the *safety of care*, the mortality rate and the safety profile of antipsychotic drug therapy (i.e. the recommended set of lab exams for evaluating the blood glucose and lipids profile to screen for the metabolic side effects of drug therapy) were assessed. The complete set of lab exams for assessing the safety profile of drug therapy was performed in about three out of ten prevalent cases treated with antipsychotics. Only one out of ten newly taken-in-care patients who started an antipsychotic drug therapy was monitored both for hyperglycaemia and hyperlipidaemia. The SMR, which was only calculated for prevalent patients with schizophrenia and related disorders, showed an increased mortality rate of 70% (95% CI 60–80%) compared to the general population.

In relation to *geographical variability*, several indicators presented a marked heterogeneity across regions, especially considering the prevalent cohort. Differences in key aspects of community care, such as access and intensity of psychosocial care (psychological treatments, psychoeducation and carers' support), were evident. A patient living in Palermo received half of the community interventions and a fourth of the psychosocial interventions compared with a patient in Emilia-Romagna. Furthermore, in terms of continuity of community care, the percentages of patients in contact with community facilities within 14 days after GHPW discharge or receiving continuously a prescription of antipsychotic therapy showed high variability between regions, although readmission rates within 7–30 days of GHPW discharge were similar. The monitoring of side effects of antipsychotic drug therapy and mortality revealed huge variations. However, for new cases, the variability between regions was lower for some indicators, such as unplanned readmissions to GHPW within 30 days, accessibility to home care and antipsychotic therapy prescription.

## Discussion

The results showed a mixed picture. For most prevalent patients, community care is accessible, continuous and moderately intensive. However, the gap in psychosocial treatments (also among regions) is relevant, both for overall and specific interventions: psychoeducation or psychological treatments are not common, and the intensity of these treatments is moderate. This is also true for specific activities addressed to patients' family members. Generally, few patients receive home care, even immediately after the discharge from GHPWs. Only one-tenth of patients are admitted to GHPWs and some dimensions of hospital care quality (e.g. readmissions within 7–30 days, LoS of more than 30 days) seem guaranteed. Residential care is more frequent than acute hospital care. Continuity of care at the community level seems achieved, as in the continuity between GHPW and CMHC within 2 weeks of hospital discharge. Most patients receive antipsychotic drugs, and polytherapy is frequent. Only a half of the patients showed a long-term persistence with antipsychotic prescriptions, and the monitoring for metabolic side effects is generally poor. Mortality shows a twofold risk with respect to the general population, as also found in a recent Italian study using health administrative databases (Berardi *et al*., 2021).

The quality of care for patients newly taken-in-care is not homogeneous. Newly taken-in-care patients present a moderate intensity of outpatient care, specifically for psychosocial interventions, low home care and continuity of community care, and a considerable level of admissions to GHPWs and residential facilities, low persistence in antipsychotic drug therapy and poor medical attention in monitoring metabolic side effects. Moreover, the care pathways for new cases are not implemented based on a structured assessment, a lack hindering care appropriateness. Conversely, some areas are well addressed: the readiness of community care after hospitalisation and the percentage of patients who receive specific psychosocial interventions is acceptable for cases newly taken-in-care.

The regional heterogeneity for the prevalent cases is high in almost all areas. Between-regions variability was quite low for newly taken-in-care patients. Accessibility to home care, the continuity between GHPWs and CMHC after discharge and the continuity of the prescription of antipsychotic therapy was relatively homogenous in the four areas. When this variability is high, its meaning could be easily read in terms of quality of mental health care. The existing low variability is more difficult to interpret. The lack of variability for new cases in terms of accessibility to home care, continuity between GHPW and CMHC, and continuity of prescription of antipsychotic therapy may mean that all the regions have the same low quality of care in these areas.

The variability in the definitions of the indicators limits the international comparability of these results; standards are still lacking for the definitions of clinical indicators (Iyer *et al*., 2016). An example concerns the continuity of care between hospital and community, as the recommended period following discharge from inpatient care may vary from 7 to 30 days in different countries (OECD, 2021). However, some comparisons for the intensity of community care may be possible. UK patients received a higher mean number of outpatient contacts (Thomas *et al*., 2018) than Italian patients and a higher proportion of patients had access to psychological interventions (NICE, 2019).

The frequency of polytherapy with antipsychotics in Italy was higher than in the UK (The Royal College of Psychiatrists, 2018) or in Spain (Orrico-Sánchez *et al*., 2020). An extensive review (Gallego *et al*., 2012) showed that it was twice the median of European studies in general, but lower than in a large Finnish survey (Tiihonen *et al*., 2019). A lower percentage of Italian patients received safety profile monitoring of antipsychotic therapy in comparison to the UK (NICE, 2019).

Concerning continuity of care, while it is not possible to find a recent comparison for the indicator of continuous community care, the percentage of patients with a community contact within 7–14 days after discharge from GHPW in Norway (OECD, 2021) was similar compared to Italy and Australia (Australian Institute of Health and Welfare, 2021) but limited to a shorter period (7 days).

Considering the frequency of unplanned GHPW readmissions within 30 days, estimates for Italy were similar to those from the USA (AHRQ, 2021), Denmark (The Danish Ministry of Health *et al*., 2018), but higher than the OECD countries median (OECD, 2021) and Scotland (Scottish Government, 2021).

Mortality, expressed in SMR, was lower in Italy than in Denmark (Lomholt *et al*., 2019), in the UK (Hayes *et al*., 2017) and in Scandinavian countries (Nordentoft *et al*., 2013).

The picture of mental health care in Italy, compared to other high-income countries, is incomplete mainly because of different indicator systems. However, it is possible to state that the delivery of psychosocial interventions is insufficient, as is the monitoring of antipsychotic side effects, while the continuity of care between general hospital and the community as a percentage of repeated admissions are in line with the countries examined. More studies are needed to understand better the reasons for the lower mortality in Italy in comparison with Northern European countries.

Finally, some critical issues should be addressed before a quality improvement of the Italian Mental Health System occurs. First, current community care is focused more on the delivery of psychiatric care and less on psychosocial-oriented care. Consistently with previous studies (Pathare *et al*., 2018; Corrao *et al*., 2021), stressing the role of the ‘psychosocial intervention gap’ (i.e. the lack of psychosocial interventions in mental health care), we strongly support the need to increase the provision of community psychosocial care in mental health services. The only sustainable way to bridge the psychosocial gap is to empower nurses, rehabilitation therapists and social workers as providers of psychosocial interventions through a wide and structural process of task shifting (WHO|Task shifting, s.d.). Second, cases newly taken-in-care receive less intensive and continuous care than prevalent ones. The implementation in Italy of Early Intervention Services using structured clinical pathways for newly taken-in-care patients is incomplete (Csillag *et al*., 2018). Addressing this deficit in community care must be prioritised. Third, efforts should be made to improve the safety of psychopharmacological treatments and, more generally, to reduce mortality. Fourth, geographical variability is a key issue, as highlighted in the USA (Horvitz-Lennon *et al*., 2015), because relevant gaps between regions are likely to hamper equity. These gaps should be addressed in terms of resources. In addition, the rate of mental health professionals among the four regions shows a twofold variation (Italian Ministry of Health, 2018), and there is need for the reorganisation of mental health services at a regional level. Although more resources are urgently needed, an increase in resources alone is not sufficient. The organisation of mental health services in different regions should be better understood and eventually changed.

To conclude this study, we need to consider its strengths and weaknesses. First, our investigation is based on data from a large, unselected cohort across four Italian regions, covering about one-fifth of the national population. This was possible because Italy's free healthcare system is available to all citizens. This study represents the largest evaluation carried out in Italy, on the quality of mental health care routinely delivered to patients affected by prevalent or incident schizophrenia. It is among the most extensive surveys conducted in a European country, comparable to studies based on the Danish Schizophrenia Register (Jørgensen *et al*., 2015, 2016; Baandrup *et al*., 2016) or other Nordic mental health registers (Nordentoft *et al*., 2013; van der Lee *et al*., 2016; Tiihonen *et al*., 2019). Second, the availability of high-quality integrated individual data (the HCU databases) on outpatient and inpatient services provided by the NHS, linked to data on care provided by public DMHs, offers the opportunity to trace and evaluate the complete care pathway of patients with severe mental disorders, in a setting reflecting the current clinical practice, thereby generating reliable real-world evidence on mental healthcare (Corrao *et al*., 2021). Therefore, the study is not affected by selective participation or recall bias. Third, newly taken-in-care patients were identified from their first contact with the NHS mental health services in which a schizophrenic disorder was diagnosed, and the complete sequence of their mental healthcare services was known.

Our study also has limitations. First, by using HCU databases, we were reasonably able to detect only the first contact with the diagnosis of schizophrenia and related disorders registered on the public Regional Health System, and not the date of the actual onset. Although we designed our study to start observation from when a patient was diagnosed with schizophrenia and related disorders, our previous findings suggested that this did not always occur (Corrao *et al*., 2021), probably due to our inability to account for private services. Thus, as the onset of schizophrenia and related disorders is usually less likely to occur after the age of 40, we used a different age range for including newly taken-in-care patients, similar to a recent survey in Spain using HCU to assess the epidemiology of schizophrenia (Orrico-Sánchez *et al*., 2020). Second, as with any observational study based on HCU databases, a pitfall of our study concerns the lack of clinical data (e.g. the severity of the disease, other related complications and/or comorbidities) and socioeconomic information, potentially affecting the adherence of patients to the mental healthcare supplied. Clinical status only can be inferred from diagnostic information, and information stored in the HCU databases does not include clinical variables. Furthermore, some indicators that were initially identified, as the rate of patients restrained in GHPWs or the existence of a case manager or an individual care plan, could not be evaluated because this information is not collected in the Italian Mental Health Information System. A deeper knowledge of clinical and social traits would have explained some of the findings more clearly. Third, information about treatments supplied by private organisations or facilities, as well as non-reimbursable payments, were not available from our databases (Pauly *et al*., 2016; Islek *et al*., 2018), and we could not account for these interventions in our analyses. Therefore, it should be underlined that our findings only focus on services supplied by public facilities. Fourth, we cannot exclude the possibility that some between-region differences are partly due to heterogeneity in the quality and completeness of data. However, it should be stressed that, because HCU data are used for reimbursing public and accredited service providers, incorrect and incomplete reports lead to legal consequences.

## Conclusion

This system of clinical indicators could be a useful tool for evaluating the quality of health care in a community-oriented mental health system, such as the Italian one, in an automated and standardised way. Indeed, because this system is completely based on a minimum set of HCU data already available in each Italian region, it could easily be implemented for rapid and periodical evaluations. For this purpose, a ‘dashboard’ with software for calculating these indicators has been developed and proposed for implementation to the MoH and to the Italian regions by the QUADIM-MAP working groups, with the aim of routinely assessing the quality of clinical pathways and providing benchmarking for DMHs at national and regional level.

In Europe, only a few countries currently use indicators to routinely assess the quality of mental health care. However, the rising interest in this field reflects the opinion that data-based and systematic quality monitoring at a regional and/or national level are essential for the sustainable improvement of mental healthcare (Bramesfeld *et al*., 2016). Real-world data collected in HCU databases may represent essential leverage for quality improvement of mental health care. These data are needed now more than ever because the COVID-19 outbreak seriously hampered the activity of mental health services and highlighted the importance of mental health care for the population (Mezzina *et al*., 2020). There is an urgent need to analyse the impact that COVID-19 had on the quality of mental health care, a need that could be met through the ongoing assessment of the indicators system presented in this study.

## Data Availability

The data that support the findings of this study are available from the Regions of Lombardy, Lazio and Emilia-Romagna, and the Province of Palermo, but restrictions apply to the availability of these data, which were used under license for the current study, and so are not publicly available. Data are however available from the authors upon reasonable request and with permission of the Regions involved in this study.

## Supplementary material

For supplementary material accompanying this paper visit https://doi.org/10.1017/S2045796022000014. click here to view supplementary material
