# Supplementary material for: The quality of mental health care delivered to patients with schizophrenia and related disorders in the Italian mental health system. The QUADIM project: a multi-regional Italian investigation based on healthcare utilisation databases
Source: Epidemiol Psychiatr Sci. 2022 Feb 14;31:e15. doi: 10.1017/S2045796022000014 (PMC8851066; doi:10.1017/S2045796022000014)
Supplement: Supplementary file 1 [file S2045796022000014sup001.docx]

**The quality of mental health care delivered to patients with schizophrenia and related disorders in the Italian mental health system. The QUADIM project**

**A multi-regional Italian investigation based on healthcare utilization databases.**

Antonio LORA^1,2^, Matteo MONZIO COMPAGNONI^2,3^, Liliana ALLEVI^1^, Angelo BARBATO^4^, Flavia CARLE^2,5^, Barbara D’AVANZO^4^, Teresa DI FIANDRA^6^, Lucia FERRARA^7^, Andrea GADDINI^8^, Melania LEOGRANDE^2,3^, Alessio SAPONARO^9^, Salvatore SCONDOTTO^2,10^, Valeria D TOZZI^7^, Simona CARBONE^11^, Giovanni CORRAO^2,3^, on behalf of the “*QUADIM project*” and “*Monitoring and assessing diagnostic-therapeutic paths (MAP)*” working groups of the Italian Ministry of Health

^1^ Department of Mental Health and Addiction Services, ASST Lecco, Lecco, Italy

^2^ National Centre for Healthcare Research and Pharmacoepidemiology, University of Milano-Bicocca, Milan, Italy

^3^ Unit of Biostatistics, Epidemiology and Public Health, Department of Statistics and Quantitative Methods, University of Milano-Bicocca, Milan, Italy

^4^ Unit for Quality of Care and Rights Promotion in Mental Health, Istituto di Ricerche Farmacologiche Mario Negri IRCCS, Milano, Italy

^5^ Center of Epidemiology and Biostatistics, Polytechnic University of Marche, Ancona, Italy

^6^ Psychologist, previously General Directorate for Health Prevention, Ministry of Health, Rome, Italy

^7^ Centre of Research on Health and Social Care Management, SDA Bocconi School of Management (Bocconi University) Milan, Italy

^8^ Agency for Public Health, Lazio Region, Rome, Italy

^9^ General Directorate of Health and Social Policies, Emilia-Romagna Region, Bologna, Italy

^10^ Department of Health Services and Epidemiological Observatory, Regional Health Authority, Sicily Region, Palermo, Italy

^11^ Department of Health Planning, Italian Health Ministry, Rome, Italy

**SUPPLEMENTARY MATERIAL**

**Address for correspondence**: Dr. Matteo Monzio Compagnoni, Division of Biostatistics, Epidemiology and Public Health, Department of Statistics and Quantitative Methods, University of Milano-Bicocca, Street Bicocca degli Arcimboldi, 8, Building U7, 20126 Milan, Italy. E-mail: [**matteo.monziocompagnoni@unimib.it**](mailto:matteo.monziocompagnoni@unimib.it)

**Supplementary Table S1.** Service interventions, treatments and activities delivered by Community Mental Health Centers (CMHCs) and Day Centers (DCs), and their classification in the Italian Mental Health Information System.

| **OUTPATIENT VISITS** (delivered by territorial and day-care facilities of CMHCs) | **Italian Mental Health Information system codes** |
| --- | --- |
|  |  |
| **Generic care** |  |
| Psychiatric visit | 01 |
| Individual meeting with a professional | 03 |
| Consultation | 04 |
| Medico-legal assessment | 05 |
| Standardized psychological assessments using test | 06 |
| Drug administration | 11 |
| Meeting with relatives | 12 |
| Staff Meeting | 15 |
| Healthcare facilities stay | 20 |
| Support to daily living activity | 24 |
| Network interventions | 26 |
| **Psychosocial interventions** |  |
| Individual living skills training | 16 |
| Group living skills training | 17 |
| Individual socialization | 18 |
| Group of socialization | 19 |
| Individual bodywork (i.e., expressive, practical manual and motor intervention), leisure activities | 21 |
| Group bodywork (i.e., expressive, practical manual and motor intervention), leisure activities | 22 |
| Vocational training | 23 |
| Assistance with financial and welfare procedures; leisure activities | 25 |
| **Psychotherapy** |  |
| Psychological visit | 02 |
| Individual psychotherapy | 07 |
| Couple psychotherapy | 08 |
| Family psychotherapy | 09 |
| Group psychotherapy | 10 |
| **Psychoeducation** |  |
| Single family psychoeducation | 13 |
| Multifamily group psychoeducation | 14 |

**Supplementary Table S2.** Diagnostic and therapeutic (ICD-9-CM, ICD-10, and ATC) codes used in the current study for drawing records and fields from Healthcare Utilization databases.

| **SCHIZOPHRENIA** | |
| --- | --- |
|  | **ICD-10 codes**  **(Lombardy)** |
| Schizophrenia | F20.* |
| Schizotypal disorder | F21.* |
| Delusional disorders | F22.* |
| Brief psychotic disorder | F23.* |
| Shared psychotic disorder | F24.* |
| Schizoaffective disorders | F25.* |
| Other psychotic disorder not due to a substance or known physiological condition | F28.* |
| Unspecified psychosis not due to a substance or known physiological condition | F29.* |
|  | **ICD-9-CM codes**  **(Emilia-Romagna, Lazio and Palermo)** |
| Schizophrenic disorders | 295.* |
| Delusional disorders | 297.* |
| Other nonorganic psychoses | 298.2, 298.3, 298.8, 298.9 |
| Psychogenic paranoid psychosis | 298.4 |
| **DRUGS** | |
|  | **ATC codes** |
| Antipsychotic agents | N05A (excluded N05AN) |
| **OUTPATIENT PROCEDURES** | |
|  | **National procedure codes** |
| Psychiatric visit | 94.12.1, 94.19.1 |
| Psychological interview | 94.09 |
| Standardized psychological assessments using test | 94.01.1, 94.08.3, 94.08.4, 94.08.5, 94.08.6 |
| Couple/Family psychotherapy | 94.3, 94.42 |
| Group psychotherapy | 94.44 |
| Glycated haemoglobin | 90.27.1, 90.28.1 |
| Lipid profile | 90.14.1, 90.14.3, 90.43.2 |

**Supplementary Table S3.** Baseline characteristics of prevalent patients with Schizophrenia and related disorders treated by DMHs of four Italian areas (Lombardy, Emilia Romagna and Lazio Regions and Province of Palermo) and in the whole sample. Italy, QUADIM-MAP projects, Italy, 2015-2016

|  | Lombardy  (N=36,076) | Emilia-Romagna  (N=17,230) | Palermo  (N=5700) | Lazio  (N=11,580) | All together  (N=70,586) |
| --- | --- | --- | --- | --- | --- |
|  |  |  |  |  |  |
| **Gender** |  |  |  |  |  |
| Men | 19,542 (54.2%) | 8888 (51.6%) | 3376 (59.2%) | 6541 (56.5%) | 38,347 (54.3%) |
| Women | 16,534 (45.8%) | 8342 (48.4%) | 2324 (40.8%) | 5039 (43.5%) | 32,239 (45.7%) |
| **Age (years)** |  |  |  |  |  |
| Mean (SD) | 51.1 (14.4) | 51.6 (14.8) | 48.8 (14.4) | 49.5 (13.8) | 50.3 (14.3) |
| 18-25 | 1341 (3.7%) | 662 (3.9%) | 310 (5.4%) | 505 (4.4%) | 2818 (4.0%) |
| 26-40 | 7069 (19.6%) | 3331 (19.3%) | 1353 (23.7%) | 2362 (20.4%) | 14,115 (20.0%) |
| 41-49 | 8628 (23.9%) | 4013 (23.3%) | 1395 (24.5%) | 3083 (26.6%) | 17,119 (24.3%) |
| ≥50 | 19,038 (52.8%) | 9224 (53.5%) | 2642 (46.4%) | 5630 (48.6%) | 36,534 (51.7%) |
| **Education years** |  |  |  |  |  |
| 0-5 | 9293 (25.8%) | 3418 (19.8%) | 1839(32.3%) | 1601 (13.8%) | 16,151 (22.9%) |
| 6-8 | 15,313 (42.4%) | 6842 (39.7%) | 3670 (64.4%) | 4793 (41.4%) | 30,618 (43.4%) |
| 9-13 | 8324 (23.1%) | 4716 (27.4%) | 0 (0%) | 3504 (30.3%) | 16,544 (23.4%) |
| ≥14 | 1329 (3.7%) | 940 (5.5%) | 186 (3.3%) | 579 (5.0%) | 3034 (4.3%) |
| *Missing data* | 1817 (5.0%) | 1314 (7.6%) | 5 (0.1%) | 1103 (9.5%) | 4239 (6.0%) |
| **Job condition** |  |  |  |  |  |
| Employed | 23,463 (65.0%) | 3608 (20.9%) | 607 (10.6%) | 2438 (21.1%) | 30,116 (42.7%) |
| Unemployed | 4156 (11.5%) | 8375 (48.6%) | 4477 (78.5%) | 8207 (70.9%) | 25,215 (35.7%) |
| Invalid | 6460 (17.9%) | 1976 (11.5%) | 611 (10.7%) | 22 (0.2%) | 9069 (12.8%) |
| *Missing data* | 1997 (5.5%) | 3271 (19.0%) | 5 (0.1%) | 913 (7.9%) | 6186 (8.8%) |
| **Family arrangement** |  |  |  |  |  |
| Living with family | 26,774 (74.2%) | 12,464 (72.3%) | 1977 (34.7%) | NA | 41,215 (69.8%) |
| Living in community | 1972 (5.5%) | 979 (5.7%) | 375 (6.6%) | NA | 3326 (5.6%) |
| Living alone | 5364 (14.9%) | 2284 (13.3%) | 352 (6.2%) | NA | 8000 (13.6%) |
| *Missing data* | 1966 (5.4%) | 1503 (8.7%) | 2996 (52.6%) | NA | 6465 (11.0%) |
| **Marital status** |  |  |  |  |  |
| Unmarried | 21,632 (60.0%) | 10,140 (58.9%) | 3588 (62.9%) | 7786 (67.2%) | 43,146 (61.1%) |
| Married | 8972 (24.9%) | 3823 (22.2%) | 1483 (26.0%) | 2119 (18.3%) | 16,397 (23.2%) |
| Separated | 1558 (4.3%) | 681 (4.0%) | 231 (4.1%) | 582 (5.0%) | 3052 (4.3%) |
| Divorced | 1402 (3.9%) | 735 (4.3%) | 122 (2.1%) | 319 (2.8%) | 2578 (3.7%) |
| Widow/er | 1182 (3.3%) | 592 (3.4%) | 191 (3.4%) | 213 (1.8.%) | 2178 (3.1%) |
| *Missing data* | 1330 (3.7%) | 1259 (7.2%) | 85 (1.5%) | 561 (4.8%) | 3235 (4.6%) |
| **Diagnosis** |  |  |  |  |  |
| F2 (Schizophrenia, schizotypal and delusional disorders) | 3417 (9.5%) | 0 (0%) | 0 (0%) | 0 (0%) | 3417 (4.8%) |
| F20 (Schizophrenia) | 14,375 (39.8%) | 7752 (45.0%) | 4107 (72.1%) | 6334 (54.7%) | 32,568 (46.2%) |
| F21 (Schizotypal disorder) | 1207 (3.3%) | 190 (1.1%) | 145 (2.5%) | 242 (2.1%) | 1784 (2.5%) |
| F22 (Delusional disorders) | 4278 (11.9%) | 3323 (19.3%) | 176 (3.1%) | 1048 (9.1%) | 8825 (12.5%) |
| F23 (Brief psychotic disorder) | 4433 (12.3%) | 1618 (9.4%) | 381 (6.7%) | 1229 (10.6%) | 7661 (10.9%) |
| F24 (Shared psychotic disorder) | 69 (0.2%) | 19 (0.1%) | 0 (0%) | 16 (0.1%) | 104 (0.1%) |
| F25 (Schizoaffective disorders) | 4352 (12.1%) | 2925 (17.0%) | 381 (6.7%) | 1644 (14.2%) | 9302 (13.2%) |
| F28 (Other nonorganic psychotic disorders) | 442 (1.2%) | 0 (0%) | 0 (0%) | 0 (0%) | 442 (0.6%) |
| F29 (Unspecified nonorganic psychosis) | 3503 (9.7%) | 1403 (8.1%) | 510 (8.9%) | 1067 (9.2%) | 6483 (9.2%) |

DMH: Department of Mental Health

**Supplementary Table S4.** Baseline characteristics of patients with Schizophrenia and related disorders newly taken-in-care by DMHs of four Italian areas (Lombardy, Emilia Romagna and Lazio Regions and Province of Palermo) and in the whole sample. Italy, QUADIM-MAP projects, Italy, 2015-2016

|  | Lombardy  (N=637) | Emilia-Romagna  (N=459) | Palermo  (N=152) | Lazio  (N=504) | All together  (N=1752) |
| --- | --- | --- | --- | --- | --- |
|  |  |  |  |  |  |
| **Gender** |  |  |  |  |  |
| Men | 429 (67.3%) | 290 (63.2%) | 112 (73.7%) | 317 (62.9%) | 1148 (65.5%) |
| Women | 208 (32.7%) | 169 (36.8%) | 40 (26.3%) | 187 (37.1%) | 604 (34.5%) |
| **Age (years)** |  |  |  |  |  |
| Mean (SD) | 29.0 (6.7) | 29.0 (6.5) | 29.5 (6.4) | 30.4 (6.5) | 29.5 (6.5) |
| 18-25 | 227 (35.6%) | 160 (34.9%) | 52 (34.2%) | 139 (27.6%) | 578 (33.0%) |
| 26-40 | 410 (64.4%) | 299 (65.1%) | 100 (65.8%) | 365 (72.4%) | 1174 (67.0%) |
| **Education years** |  |  |  |  |  |
| 0-5 | 252 (39.6%) | 24 (5.2%) | 19 (12.5%) | 31 (6.2%) | 326 (18.6%) |
| 6-8 | 30 (4.7%) | 168 (36.6%) | 129 (84.9%) | 202 (40.1%) | 529 (30.2%) |
| 9-13 | 152 (23.9%) | 161 (35.1%) | 0 (0%) | 175 (34.7%) | 488 (27.9%) |
| ≥14 | 134 (21.0%) | 43 (9.4%) | 3 (2.0%) | 32 (6.3%) | 212 (12.1%) |
| *Missing data* | 69 (10.8%) | 63 (13.7%) | 1 (0.7%) | 64 (12.7%) | 197 (11.2%) |
| **Job condition** |  |  |  |  |  |
| Employed | 370 (58.1%) | 107 (23.3%) | 14 (9.2%) | 106 (21.0%) | 597 (34.1%) |
| Unemployed | 85 (13.3%) | 252 (54.9%) | 134 (88.2%) | 342 (67.9%) | 813 (46.4%) |
| Invalid | 105 (16.5%) | 4 (0.9%) | 3 (2.0%) | 0 (0%) | 112 (6.4%) |
| *Missing data* | 77 (12.1%) | 96 (20.9%) | 1 (0.7%) | 56 (11.1%) | 230 (13.1%) |
| **Family arrangement** |  |  |  |  |  |
| Living with family | 491 (77.1%) | 351 (76.5%) | 70 (46.1%) | NA | 912 (73.1%) |
| Living in community | 34 (5.3%) | 19 (4.1%) | 3 (2.0%) | NA | 56 (4.5%) |
| Living alone | 50 (7.8%) | 25 (5.4%) | 3 (2.0%) | NA | 78 (6.2%) |
| *Missing data* | 62 (9.7%) | 64 (13.9%) | 76 (50.0%) | NA | 202 (16.2%) |
| **Marital status** |  |  |  |  |  |
| Unmarried | 461 (72.4%) | 322 (70.2%) | 125 (82.2%) | 406 (80.6%) | 1314 (75.0%) |
| Married | 100 (15.7%) | 64 (13.9%) | 19 (12.5%) | 42 (8.3%) | 225 (12.8%) |
| Separated | 12 (1.9%) | 9 (2.0%) | 1 (0.7%) | 10 (2.0%) | 32 (1.8%) |
| Divorced | 5 (0.8%) | 6 (1.3%) | 0 (0%) | 1 (0.2%) | 12 (0.7%) |
| Widow/er | 2 (0.3%) | 0 (0%) | 0 (0%) | 1 (0.2%) | 3 (0.2%) |
| *Missing data* | 57 (8.9%) | 58 (12.6%) | 7 (4.6%) | 44 (8.7%) | 166 (9.5%) |
| **Diagnosis** |  |  |  |  |  |
| F2 (Schizophrenia, schizotypal and delusional disorders) | 66 (10.4%) | 0 (0%) | 0 (0%) | 0 (0%) | 66 (3.8%) |
| F20 (Schizophrenia) | 121 (19.0%) | 83 (18.1%) | 42 (27.6%) | 197 (33.1%) | 413 (23.6%) |
| F21 (Schizotypal disorder) | 19 (3.0%) | 5 (1.1%) | 7 (4.6%) | 5 (1.0%) | 36 (2.0%) |
| F22 (Delusional disorders) | 51 (8.0%) | 112 (24.4%) | 6 (3.9%) | 55 (10.9%) | 224 (12.8%) |
| F23 (Brief psychotic disorder) | 162 (25.4%) | 128 (27.9%) | 53 (34.9%) | 109 (21.6%) | 452 (25.8%) |
| F24 (Shared psychotic disorder) | 6 (1.0%) | 1 (0.2%) | 0 (0%) | 2 (0.4%) | 9 (0.5%) |
| F25 (Schizoaffective disorders) | 25 (3.9%) | 29 (6.3%) | 12 (7.9%) | 62 (12.3%) | 128 (7.3%) |
| F28 (Other nonorganic psychotic disorders) | 13 (2.0%) | 0 (0%) | 0 (0%) | 0 (0%) | 13 (0.7%) |
| F29 (Unspecified nonorganic psychosis) | 174 (27.3%) | 101 (22.0%) | 32 (21.1%) | 104 (20.7%) | 411 (23.5%) |

DMH: Department of Mental Health
